# Editorial: Clinical teaching and practice in intensive care medicine and anesthesiology

**DOI:** 10.3389/fmed.2023.1207363

**Published:** 2023-06-07

**Authors:** Pan Pan, Matthieu Komorowski, Le Shen, Lgnacio Martin-Loeches, Longxiang Su

**Affiliations:** ^1^College of Respiratory and Critical Care Medicine, Eighth Medical Center, Chinese People's Liberation Army General Hospital, Beijing, China; ^2^Department of Surgery & Cancer, Imperial College London, London, United Kingdom; ^3^Department of Anesthesiology, State Key Laboratory of Complex Severe and Rare Diseases, Peking Union Medical College Hospital, Peking Union Medical College and Chinese Academy of Medical Sciences, Beijing, China; ^4^Department of Intensive Care Medicine, Multidisciplinary Intensive Care Research Organization (MICRO), St. James's Hospital, Dublin, Ireland; ^5^Department of Critical Care Medicine, State Key Laboratory of Complex Severe and Rare Diseases, Peking Union Medical College Hospital, Peking Union Medical College and Chinese Academy of Medical Sciences, Beijing, China

**Keywords:** intensive care medicine, anesthesiology, teaching, practice, technology

This special topic is a Research Topic opened by Frontiers in Medicine in the field of critical care medicine and anesthesiology, focusing on the research and discussion of clinical teaching and practice. The main purpose of this Research Topic is to promote the development of teaching and practice in the field of critical care medicine and anesthesiology, improve the teaching and practice level of medical staff, so as to provide safer and more effective medical services for critically ill patients. This Research Topic finally contains 16 articles in total, 11 of which are original articles. The studies covers many research areas related to clinical teaching and practice, including but not limited to: education system and teaching methods of critical care medicine and anesthesiology, teaching quality assessment and improvement, clinical skills training and practical operation standards, Clinical research and translational research related to critical care medicine and anesthesia, as well as clinical case analysis and study protocol.

First of all, this Research Topic contains many researches and reflections on critical care medicine and anesthesiology education. O'Connor and Doyle reviewed methods for the assessment of undergraduate clinical placements in anesthesia and critical care. The results of the study show that there are various evaluation methods, including scoring sheets, questionnaires, reflection logs, etc., but these methods lack consistency and comparability and need further improvement and unification. Only adequate assessment will lead to an appropriate educational program for the residents to improve their skills and confidence in critical situations (O'Connor and Doyle). Su et al. conducted a survey on the training status of analgesia and sedation in China. The study found the necessity of improving physicians' cognition and practice of analgesia and sedation, and provided a reference for improving related education and training in mainland China (Su et al.). Brewster et al. the leadership in airway management needs to be further strengthened, and at the same time, it is necessary to pay attention to the teamwork ability in the airway management process (Su et al.). In addition, the Research Topic also introduced the development and training of palliative medicine in China, opened up the extension of intensive care medicine treatment, and explained the possible methods of how to realize palliative care in intensive care medicine (Brewster et al.). These articles highlight the critical role of education in improving the quality and safety of critical care healthcare and provide physicians with educational resources on best practices and new technologies.

Second, this Research Topic contains several articles related to the diagnosis and management of critically ill patients. Among them is an analysis of septic shock in plateau areas from China. Studies have explained that septic shock is mainly caused by pathogens such as pneumonia, abdominal infection, and urinary tract infection, and has a high prevalence rate. In addition, patients with septic shock at high altitude often have challenges in airway management, hemodynamics, and fluid management (Li Q. et al.). This clarifies the direction of future related plateau medical training. In addition, severe disease-related technologies are developing rapidly, and two studies are from Long's Team. Their case report on the diagnosis of massive atelectasis and pneumothorax using electrical impedance imaging demonstrated the powerful diagnostic power of EIT (Zhou et al.); another article described a standard method for esophageal pressure measurement (Jiang et al.), which is relevant Training and dissemination of technology is critical care. In addition, it is of great significance to improve the treatment of patients who cannot tolerate non-invasive mechanical ventilation. Altinkaya Cavus et al. revealed the appropriate sedative agent to effectively reduce discomfort and improve the therapeutic effect, breaking through the past situation where we did not dare to use sedation for related patients (Altinkaya Cavus et al.). These studies provide important information on how to quickly and accurately diagnose and treat diseases in critical situations, helping doctors better understand how to respond to critical situations.

In addition, this Research Topic includes diagnostic and evaluation studies, articles aimed at improving the quality and safety of critical care healthcare. Among them, Mahmoodpoor et al. explored the prognostic value of using the National Early Warning Score (NEWS) and the Modified Early Warning Score (MEWS) for readmission and death in intensive care unit (ICU) patients. The conclusion highlighted that MEWS performed better in predicting death in ICU patients (Mahmoodpoor et al.). Chen et al. established an early warning scoring system for the assessment and prediction of septic shock in patients with gastrointestinal perforation. It can be seen that the evaluation of critically ill patients is very important clinically, and the scoring system needs to be paid attention to in teaching. These articles provide practical advice to help physicians refine and improve their practice in clinical setting.

Due to the characteristics of the anesthesia profession itself, this Research Topic contains many articles about the clinical practice of perioperative anesthesia. Benefits of paraspinal nerve block anesthesia (Fei et al.), fentanyl can improve poor outcome of ProSeal™ laryngeal mask insertion (Rahmat Ameen Noorazyze et al.), use of tapered cuff endotracheal tube in anterior cervical spine surgery can control air leaks and complications (Li Y-S. et al.), Sumodexin to reduce the incidence of postoperative nausea and vomiting (Mat et al.), spinal analgesia in cesarean section provides a more accurate reference for the use of bupivacaine dosage (Manouchehrian et al.). These results are of great significance to the teaching of anesthesia.

The teaching of intensive care and anesthesia is a long-term and arduous systematic project, and good training and education directly affect the quality of patient treatment. Through this Research Topic, we feel that we should continue to strengthen our understanding and investment in teaching, and constantly adjust teaching methods and directions according to clinical needs, so as to respond to changes without change.

## Author contributions

PP wrote this article. MK, LS, LM-L, and LS contributed the idea of this topic. All authors contributed to the article and approved the submitted version.

